# Waterhouse-Friderichsen syndrome following a dog bite in an asplenic patient: case report and review of the literature

**DOI:** 10.1186/s12245-023-00483-3

**Published:** 2023-02-23

**Authors:** Giorgio Berlot, Ariella Tomasini, Silvia Zanchi, Edoardo Moro, Maurizio Pinamonti, Sara Mohamed, Eleonora de Bellis

**Affiliations:** 1 Department of Anesthesia and Intensive Care, Azienda Sanitaria Universitaria Integrata Di Trieste, Trieste, 34149 Italy; 2 Department of Pathology, Azienda Sanitaria Universitaria Integrata Di Trieste, Trieste, Italy; 3 Department of Hematology, Azienda Sanitaria Universitaria Integrata Di Trieste, Trieste, Italy

**Keywords:** Waterhouse-Friderichsen syndrome, Septic shock, Disseminated intravascular coagulation, *Capnocytophaga canimorsus*

## Abstract

The Waterhouse-Friderichsen syndrome represents a critical condition characterized by a septic shock associated with a disseminated intravascular coagulation causing the plugging of the microvascular network virtually all organs and systems, including the skin, the kidneys, the liver, and adrenal glands; the mortality rate is elevated, and survivors often must undergo multiple limb amputations. Here, we describe the uncommon case of an asplenic patient who developed this syndrome after a superficial wound caused by a dog bite causing an initial infection due to *Capnocytophaga canimorsus* that is part of the normal oral microbiome of pets. The clinical and pathological findings and the current and future therapeutic options are reviewed and discussed.

## Background

The clinical trajectories of septic shock are extremely variable, ranging from cases promptly responding to the therapy to fulminant forms rapidly evolving toward a multiple organ dysfunction syndrome (MODS) and death. Recently, Kyriazopoulou et al. [1] identified a subgroup of high-mortality septic shock patients presenting the triad of hepatobiliary dysfunction (HBD), disseminated intravascular coagulation (DIC), and reactive hemophagocytic syndrome (rH) as indicated by an elevated HScore [2] and denominated this condition as macrophage activation-like syndrome (MALS); similarly to what has been described in other septic shock-like syndromes, the responsible mechanism consists in an inflammatory reaction that appears disproportionate to underlying infection [3].

The Waterhouse-Friderichsen syndrome, which is an uncommon and often fatal kind of septic shock characterized by the bilateral adrenal hemorrhage, fulfills the diagnostic criteria of MALS due to its stormy clinical course and the involvement of all organs and systems; although it has been initially associated with Neisseria infections, several other bacterial and viral strains can cause it [4]; among them, *Capnocytophaga canimorsus*, which is part of the normal oral microbiome in as many as 40% of cats and dogs [5], appears to play a peculiar role as even small skin lacerations infected by this germ can cause a rapidly evolving MODS either in patients with reduced immune capabilities or albeit more uncommonly in immunocompetent hosts [6–8].

Here, we describe the case of a patient who developed a MALS and a Waterhouse-Friderichsen syndrome following a minor wound caused by a dog bite.

## Case presentation

A 53-year-old woman was bitten on her right wrist by a dog. The remote history revealed a splenectomy due to trauma 23 years prior the current admission. As the wound appeared superficial, she seeked medical attention at the local emergency department (ED) only 3 days later when the site became reddish and painful; after the cleansing of the wound, she was discharged home with a prescription of an oral macrolide three times/day. On the morning of the 2nd after this visit, her husband took her to the hospital again as she was confused and many petechiae appeared on her trunk and upper limbs; after a short stay in the ED, she was admitted to our intensive care unit (ICU) with the diagnosis of septic shock associated with a DIC.

**Fig. 1:**
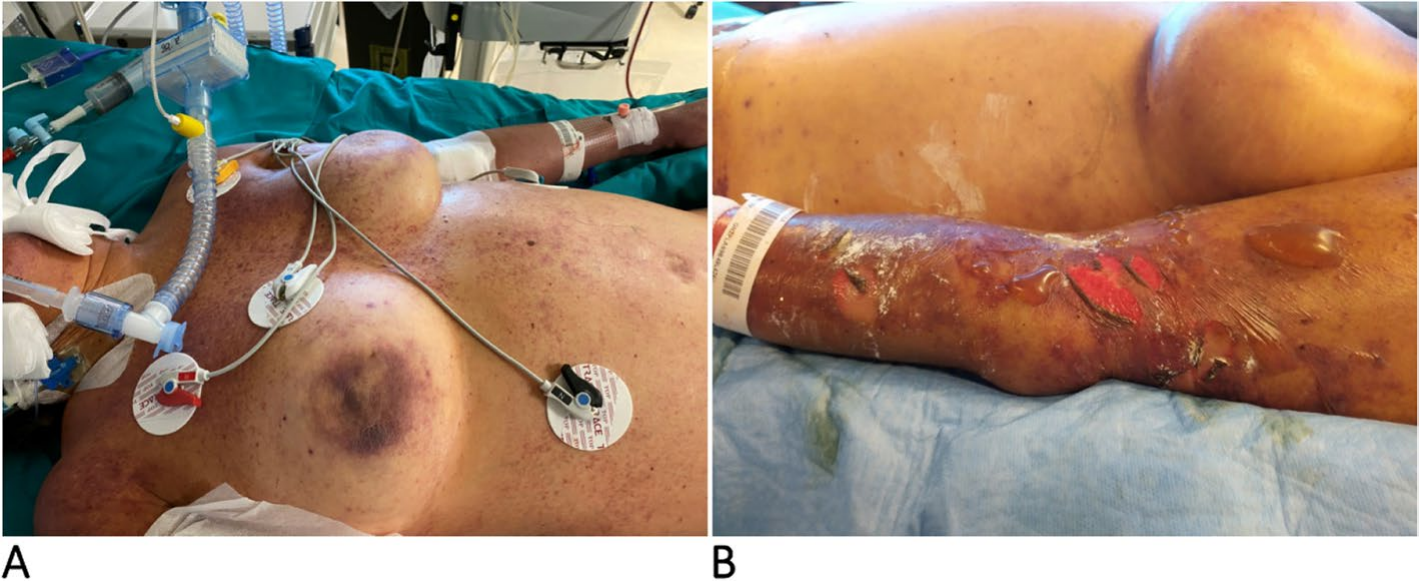
**A** Admission: multiple petechiae on the trunk and upper limbs. **B** Diffuse purpura evolving in blisters and exfoliated areas. The picture was taken 24 h after that of **A**

**Fig. 2:**
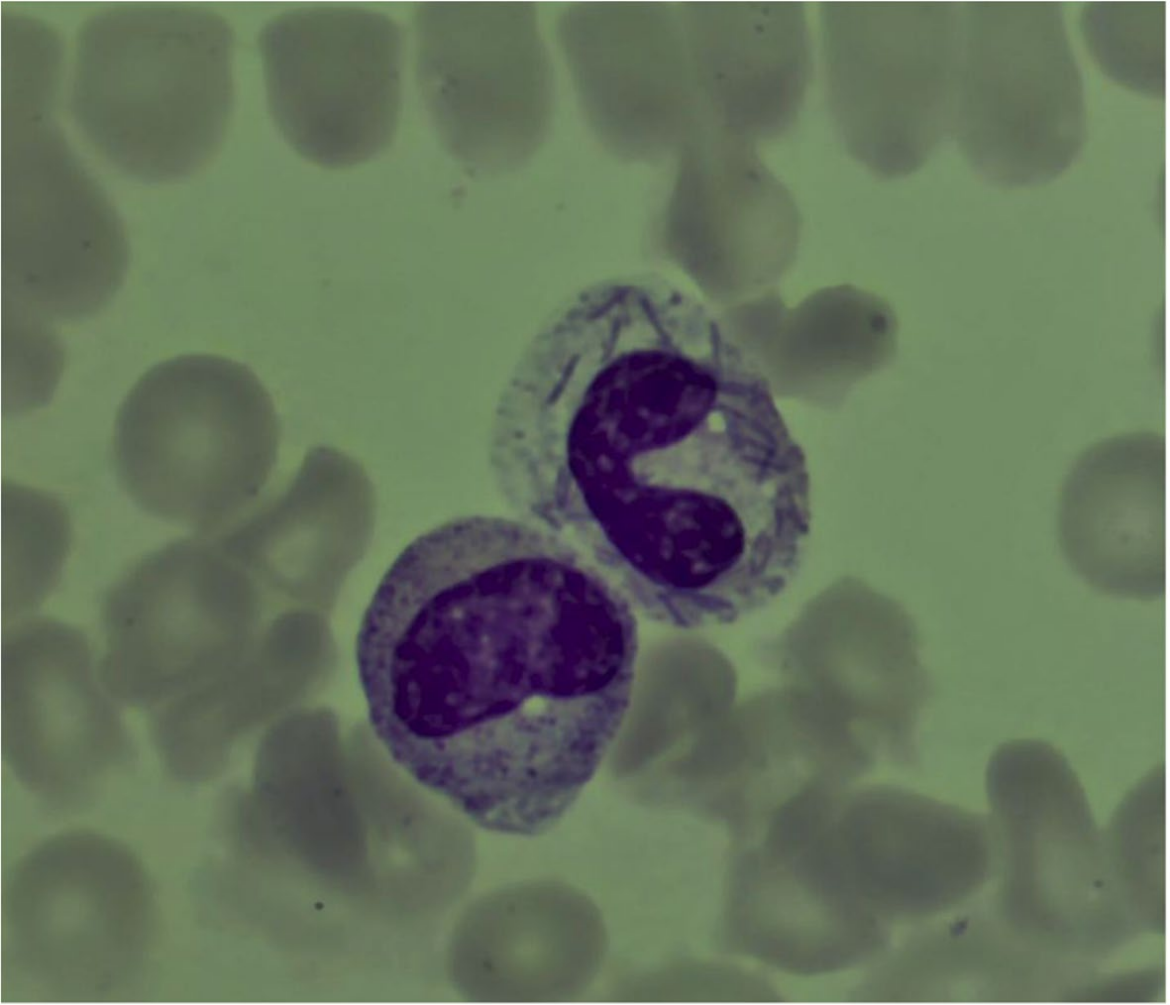
Neutrophils with intracytoplasmic bacilli

**Fig. 3:**
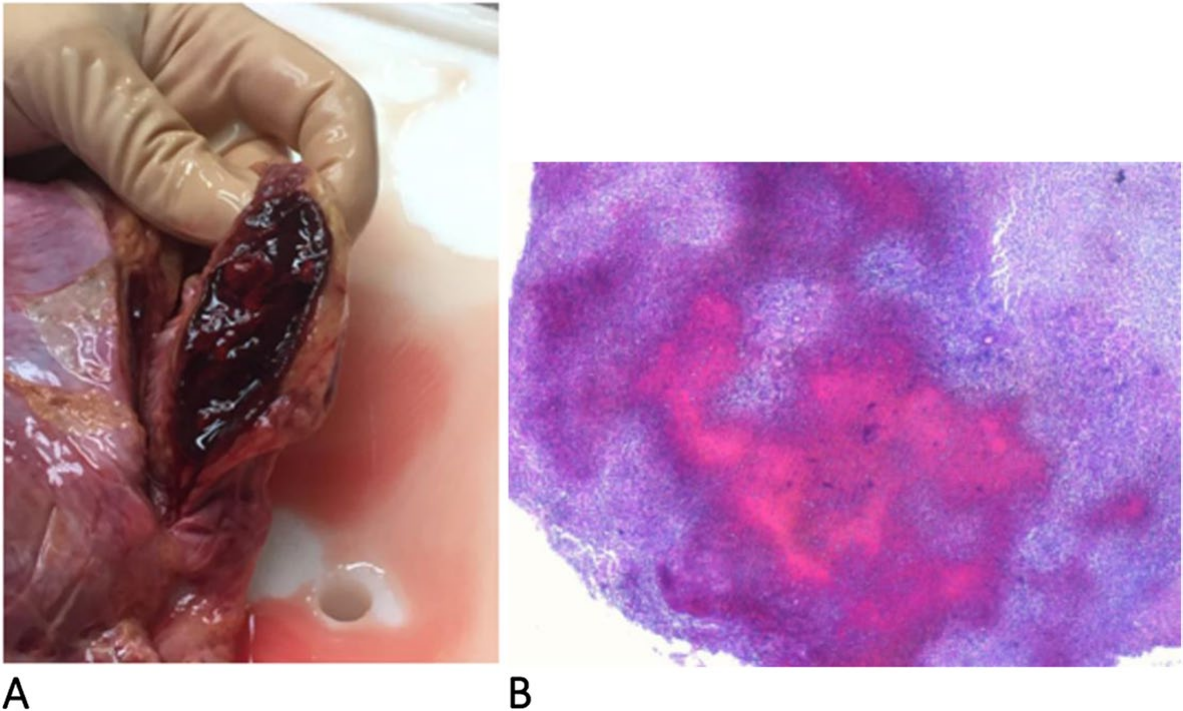
Adrenal hemorrhage. **A** At the autopsy. **B** Intraglandular large hemorrhagic and necrotic areas (H&E, 2.5*)

**Fig. 4:**
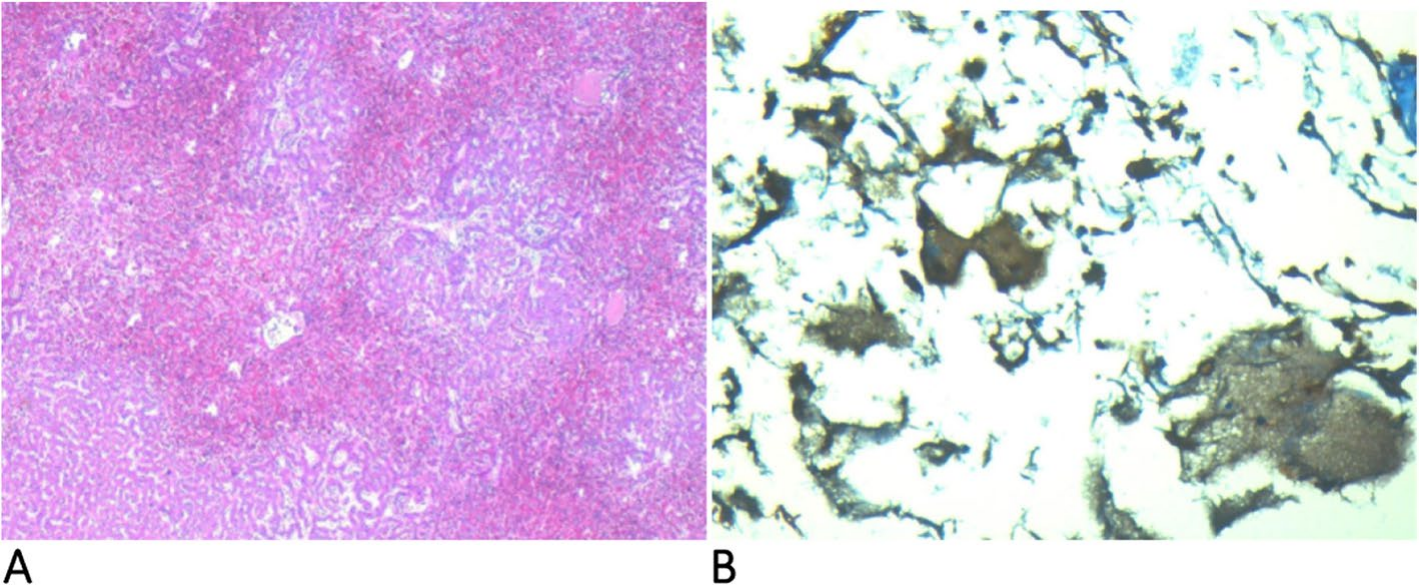
**A** Large areas of intense blood stasis and diffuse ischemia of the liver parenchyma (H&E, 2.5*). **B** Some aspects of hemophagocytosis in the bone marrow (CD163 *20)

At the clinical examination, the patient was hypotensive (80/50 mmHg) and drowsy but still arousable; the torso and the upper limbs presented multiple hemorrhagic petechiae that in the following day extended to the whole body, evolving in blisters that subsequently exfoliated (Fig. 1 A and B, respectively); as the hypotension was not responsive to the fluid resuscitation, a continuous infusion of norepinephrine was started at incremental dosages associated with iv steroids given initially as repeated boluses and as a continuous infusion later on. The blood chemistries and the coagulative tests indicated a severe hyperlactatemia and associated with HBD, acute kidney injury (AKI), and DIC (Table 1). The patient was intubated and mechanically ventilated: iv, gentamicin, ceftriaxone, and clindamycin were started along with an IgA- and IgM-enriched intravenous immunoglobulin preparation (eIg) (Pentaglobin®, Biotest, Dreiech, Germany). A continuous veno-venous hemodiafiltration (CVVHD) coupled with hemoperfusion (HP) with CytoSorb® (Cytosorbents Corporation, New Jersey, USA; Aferetica s.r.l. Bologna, Italy) was started in order to treat the AKI and to remove the septic mediators; blood samples were drawn for cultures, and a peripheral blood smear demonstrated gram bacilli inside the cytoplasm of neutrophils (Fig. 2). During the admission, the HBD, the AKI, and the DIC worsened despite the aggressive treatment, and the repeated administration of fresh-frozen plasma (FFP) and antithrombin III concentrates to provide anti-coagulative factors; the transcutaneous oximetry (TcPO2) indicated the absence of subcutaneous blood flow in all limbs despite the presence of peripheral pulses. According to the H-score, the probability of a rH was > 95% 2. On the ICU 4th, the patient became unresponsive to the vasopressors, and, in agreement with her relatives, the treatment was shifted to comfort measures only. At the autopsy, a bilateral adrenal hemorrhagic necrosis was demonstrated (Fig. 3A and B); the liver was ischemic, and the bone marrow was hypoplasic with areas of hemophagocytosis (Fig. 4A and B, respectively). Notably, three weeks after the admission the blood cultures remained negative.

**Table 1:** Time course of blood chemistries and coagulative parameters. In case of repeated measurements, the worst values for each day are reported

**Variables**	**RV**	**D1 (admission)**	**D2**^**a**^	**D3**^**a**^	**D4**^**a**^
**Hb (g/dl)**	12–16	10.8	8.6	8.3	9.2
**RBC (**^**a**^**106/ml)**	4.2–5.0	3.44	2,750	2,650	4,920
**WBC (**^**a**^**103/ml)**	4.0–11.0	1.90	15.4	32.65	55.60
**Platelets (**^**a**^**103/ml)**	150–450	8.0	14.2	15.1	8.0
**INR**	0.78–1.20	Nc	7.23	3.79	4.03
**APTTr**	0.76–1.18	Nc	2.5	3.95	3.94
**Fibrinogen (mg/dl)**	180–360	118	68	120	109
**Antithrombin III (%)**	70–110	25	13	27	23
**D-dimer (ng/ml FEU)**	< 500	42.946	112.057	126.574	102.117
**pH**	7.38–7.42	7.06	7.12	7.25	7.13
**HCO3** − **(mEq/l)**	23–25	8.9	12.3	21.0	20.7
**Glycemia (mg/dl)**	65–110	54	122	118	86
**Tryglicerides (mg/dl)**	< 170	96	Na	Na	300
**Lactate (mg/dl)**	6.5–19.3	191	157	127	106
**Creatinine (mg/dl)**	0.4–1.1	2.61	2.21	1.73	1.68
**BUN (mg/dl)**	15–50	134	41	38	40
**Creatin kinase (U/l)**	25–195	Na	525	7713	15,400
**AST/ALT (U/l)**	< 35/ < 35	3193/2278	7956/4308	11,197/5089	7308/4194
**NH4 + (µg/dl)**	27–90	200	279	533	817
**Total bilirubin (mg/dl)**	0.30–1.20	2.71	2.36	3.02	3.05
**Ferritin (µg/l)**	10–280	3773	Na	Na	> 7500
**Procalcitonin µg/l**	< 0.5	Na	28.56	14.77	7.13
**PCR (mg/l)**	< 5	141	51.3	75	112

*Legend*: *RV* range values, *nc* not coagulable, *na* not available, *INR* international normalized ratio, *APTT* activated plasma thromboplastin time ratio

^**a**^During CVVHD and HA

## Discussion

In asplenic and/or immunodepressed patients, the infection caused by *C. canimorsus* can determinate a Waterhouse-Friderichsen syndrome evolving in a MODS [4]. The clinical course is characterized by a septic shock complicated by a DIC that is responsible of the diffuse microvascular thromboses accounting for (a) the rapidly extending skin infarctions 9, (b) the occurrence of HBD and AKI, and (c) the adrenal failure caused by the plugging of the adrenal venules draining the medullary sinusoids and the subsequent increase of the venous pressure leading to the intraglandular hemorrhage. The rapid deterioration of the clinical conditions alongside with the widespread involvement of the skin accounts for the denomination of “purpura fulminans” (PF) that is often used as a synonymous of the syndrome [9–14]. The underlying causes are not fully understood and include (a) the capsular polysaccharide that affects the host–pathogen interaction by blocking the complement and the phagocytosis, (b) the presence of catalase making them resistant to the H2O2 produced by the macrophages, and (c), in asplenic subjects, the lack of an adequate number of B1a cells due to the absence of the splenic marginal zone B [10].

In our patient, although the cultures remained negative after 3 weeks of incubation, the MALS can be likely ascribed to a *C. canimorsus* infection due to the asplenia, the recent dog bite, the stormy course, and the gram bacilli visible inside the neutrophils that were identical to those described in other cases of septic shock due to same organism [14]: thus, it is conceivable that this finding could be ascribed either to the antibiotic treatment initiated some days before the ICU admission and/or to the use of a medium not allowing the growth of this germ [15].

Actually, in ours as in other cases of *C. canimorsus*-related septic shock, the MODS was determined by the highly prothrombotic DIC; although the trigger(s) are not precisely identified due to the unusual occurrence of this infection, it is has been hypothesized that the pathogen adhesion to the endothelium causes the upregulation of adhesion molecules and the margination of polimorphonucleates whose proteases impair the endothelial thrombomodulin that, in turn, substantially decreases the function of the proteins C (PC). This is the same mechanism occurring in patients with meningococcal PF [16, 17].

AS different circumstances account for the poor outcome occurred in ours as well in other patients with MALS due to *C. canimorsus*, some considerations for the treatment are warranted.

First, our patient presented a full-blown MODS already at the admission; then, since most of the treatments indicated in the Surviving Sepsis Campaign are time dependent, they could hardly modify the clinical course in an advanced phase of the disease. Consequently, patients with known risk factors for septic shock due to *C. canimorsus* should be encouraged to seek immediate medical advice in the event of a pet bite.

Second, the high mortality of *C. canimorsus*-associated MALS can be ascribed more to the occurrence of DIC than to the infection by itself; consequently, measures aiming to restore the anticoagulant capabilities, including the administration of FFP and AT III, should be adopted; the administration of PC zymogen concentrates could be considered, but the reduced or absent levels of thrombomodulin might prevent its activation.

Third, the extremely elevated CK despite the presence of peripheral pulses values likely indicates a diffuse muscle necrosis due to the DIC-associated plugging of the microvascular network despite the presence of peripheral pulses [18] it is arguable that had the patients survived, multiple amputations would have been required.

Finally, as it appears that most patients with *C. canimorsus* septic shock fail to respond to the current treatments, other approaches are required to abate the hyperinflammatory reaction that is the primer of the DIC; recently, Shakoory et al. [19] reviewed the results of a trial in which septic shock patients were given a blocker of the Interleukin-1 cellular receptor and demonstrated that only patients with MALS took advantage of this treatment; moreover, similar positive effects of Anakinra and of the Il-6 inhibitor tocilizumab have been reported also in different trials involving critical patients with severe Covid-19 pneumonia [20]. Then, the early off-label use of these substances could be valuable in patients with severe C. canimorsus infections possibly in association with HP and/or other blood purification techniques.

In our patient, all the therapeutic options that included both current and adjunctive treatment of septic shock failed to influence the clinical course likely due to the advanced phase of the disease. Patients with known risk factors for C. canimorsus infection and subsequent complications should not be discharged home but admitted to the hospital for a closer clinical survey and a prompt referral to the ICU.

## Data Availability

All data and materials are freely available from the corresponding author on reasonable request.
